# Potentiation of Recombinant NP and M1-Induced Cellular Immune Responses and Protection by Physical Radiofrequency Adjuvant

**DOI:** 10.3390/vaccines9121382

**Published:** 2021-11-24

**Authors:** Yibo Li, Zhuofan Li, Yiwen Zhao, Xinyuan Chen

**Affiliations:** Biomedical and Pharmaceutical Sciences, College of Pharmacy, University of Rhode Island, Kingston, RI 02881, USA; yibo_li@uri.edu (Y.L.); zhuofan_li@uri.edu (Z.L.); yiwen_zhao@uri.edu (Y.Z.)

**Keywords:** radiofrequency, NP, M1, cross-presentation, adjuvant, influenza

## Abstract

Nucleoprotein (NP) and matrix protein 1 (M1) are highly conserved among influenza A viruses and have been attractive targets to develop vaccines to elicit cross-reactive cytotoxic T lymphocytes (CTLs). Yet, external antigens are often presented on major histocompatibility complex class II molecules and elicit humoral immune responses. In this study, we present a physical radiofrequency adjuvant (RFA) to assist recombinant NP and M1 to elicit potent CTL responses. We found recombinant NP/M1 immunization in the presence of RFA could elicit potent anti-NP CTLs and confer significant protection against homologous viral challenges, while NP/M1 immunization alone failed to elicit significant CTL responses or confer significant protection. Interestingly, RFA failed to elicit potent anti-M1 CTL responses or anti-NP or anti-M1 antibody responses. Different from RFA, AddaVax adjuvant was found to significantly increase NP-specific antibody responses but not CTLs. NP/M1 immunization in the presence of RFA or AddaVax similarly reduced body weight loss, while only the former significantly increased the survival. We further found NP/M1 immunization in the presence of RFA did not significantly increase serum IL-6 release (a systemic inflammatory mediator) and rather reduced serum IL-6 release after boost immunization. NP/M1 immunization in the presence of RFA did not induce significant local reactions or increase body temperature of mice. The high potency and safety strongly support further development of RFA-based recombinant NP/M1 vaccine to elicit cross-protective immunity.

## 1. Introduction

Vaccine remains the most effective and cost-effective means to control influenza [1]. Currently approved influenza vaccines mainly stimulate strain-specific humoral immune responses against surface antigen hemagglutinin (HA) and are ineffective to protect against strains that have undergone antigenic drifts or shifts [1,2]. As such, current influenza vaccines need to be manufactured and immunized annually to provide updated protection against potentially different circulating strains [1,2]. Moreover, the current influenza vaccines are expected to be ineffective against the emergence of a pandemic viral strain [1,2]. Recently, universal influenza vaccines targeting conserved influenza internal antigens attracted significant attention and a number of universal influenza vaccine candidates based on internal antigens are under active development [3]. These types of vaccines mainly induce cytotoxic T lymphocytes (CTLs) to confer cross-protection [4]. 

Nucleoprotein (NP) and Matrix 1 (M1) are attractive targets for universal influenza vaccine development [5]. Studies found that M1 and NP are the immunodominant targets of cross-reactive CD4^+^ and CD8^+^ T cells against H5N1 virus in human individuals after seasonal influenza A virus infection [6]. NP has been widely explored as universal influenza vaccine antigens. Influenza NP gene has been inserted into viral vectors, such as adenovirus 5 (Ad5) [7] and chimpanzee adenovirus simian adenovirus 24 (AdC7) [8], to develop universal influenza vaccines. Besides viral vector vaccines, NP mRNA vaccines have been also under development to elicit cross-protective immunity [9]. Viral vector or mRNA-based vaccines induce NP expression in host cells, which is then presented on major histocompatibility complex (MHC) class I molecules for elicitation of NP-specific CTL responses [7,8,9]. NP-specific CTLs eliminate virus-infected cells, reduce disease severity, and promote recovery. Induction of CTL responses against multiple antigens has been an attractive approach to increase the breadths of protection [10,11]. In fact, viral vector vaccines targeting both NP and M1 have been also explored to induce cross-protective immunity. One study found NP/M1-inserted replication-defective Simian Adenovirus Vector (PanAd3) vaccine could elicit strong antibody and T-cell responses and confer protection against high-dose lethal viral challenges [12]. In another study, NP/M1-inserted replication-deficient adenovirus and modified vaccinia virus (MVA) vaccines were developed [13]. In this study, different immunization routes and modification of vaccine use in prime and boost immunizations were explored to elicit potent CTL responses against heterologous viral challenges [13].

Besides the novel types of viral vector and mRNA-based vaccines, incorporation of vaccine adjuvants into traditional protein-based vaccines may also elicit vaccine-specific CTL responses and confer cross-protection. Considering protein-based vaccines are mainly presented on MHC class II molecules and elicit humoral immune responses [14], the candidate adjuvants would need to shift the presentation of protein antigens on MHC I molecules and induce cross-presentation. Due to the limited number of vaccine adjuvants to meet vaccine development needs and the slow pace to develop chemical adjuvants [15,16,17], we took a different approach to develop physical radiofrequency (RF) adjuvant (RFA) to boost vaccination [18]. Physical RFA emits high-frequency electromagnetic waves on skin surface and causes local thermal stress with potential release of damage-associated molecular patterns (DAMPs) to alert innate immune systems to boost vaccination. Physical RFA was found to elicit transient low-level local inflammation, while chemical adjuvants were found to induce more significant local reactions in murine models [18]. Physical adjuvants are also less likely to induce significant systemic or long-term side effects considering no foreign materials enter the body. 

Our recent studies found non-invasive RF treatment of the mouse skin followed by intradermal (ID) delivery of model antigen ovalbumin (OVA) or influenza pandemic 2009 H1N1 vaccine could elicit potent humoral immune responses and at the same time induce OVA and recombinant HA (rHA)-specific CTL responses via induction of cross-presentation of protein antigens [18]. Furthermore, OVA-specific CTL responses induced by ID OVA immunization in the presence of RFA conferred significant protection against OVA-expressing E.G7 lymphoma growth in murine models [18]. This study explores whether RFA could elicit potent CTL responses against recombinant NP and M1 protein vaccines and confer protection against influenza viral challenges in murine models.

## 2. Materials and Methods

### 2.1. Reagents

Recombinant NP (11675-V08B) and M1 (40010-V07E) of influenza A/Puerto Rico/8/34/Mount Sinai (H1N1) expressed by the baculovirus-insect cell expression system were purchased from Sino Biological US Inc. (Wayne, PA, USA). AddaVax (a formulation similar to MF59 for preclinical research use) was purchased from InvivoGen (San Diego, CA, USA). Fluorescence-conjugated antibodies were purchased from BioLegend (San Diego, CA, USA).

### 2.2. Mice

C57BL/6 mice (6 weeks old, male) were purchased from Charles River Laboratories (Wilmington, MA, USA). Animals were housed in animal facilities of University of Rhode Island (URI) and anesthetized for hair removal, RF treatment, and immunization. Animal experiments involving influenza viruses were conducted in animal biosafety level 2 (ABSL2) facility of URI. All animal procedures were approved by the Institutional Animal Care and Use Committee of URI.

### 2.3. RF Device

A cosmetic fractional bipolar RF device equipped with 12 × 12 array of microelectrodes in 2 × 2 cm^2^ area was used as in our previous report [18]. This device has three energy settings (low, medium, high) and high-energy setting was used in this study to induce significant tissue stress after 1–2 min treatment. For RF treatment, a thin layer of ultrasound gel (03–08, Parker Laboratories, Fairfield, NJ, USA) was applied on the skin surface as recommended by manufacturer and RF device was then firmly pressed to allow treatment tips to have a close contact with skin surface.

### 2.4. Immunization

Hair on the lateral dorsal skin of mice was shaved and completely removed with the help of a hair removal lotion (Nair) as shown in our previous report [19]. Next day, hair-free skin was exposed to RF or sham treatment followed by ID injection of a mixture of 5 μg NP and 5 μg M1 in 20 µl (endotoxin level <1.0 EU per µg of protein) into RF or sham-treated skin or ID injection of 20 µl PBS to serve as control. Mice were also intramuscularly injected with the same amount of NP and M1 in the presence of AddaVax adjuvant (1:1 volume ratio, total 40 µl) in the thigh muscle of the hind leg. Commercial NP and M1 were dialyzed against sterile PBS for use in immunization studies. Mice were boost immunized 3 weeks later as in prime immunization. 

### 2.5. Antibody Titer Measurement

Serum antibody titer was measured by enzyme-linked immunosorbent assay (ELISA) as in our previous report [19]. In detail, ELISA plates were coated with NP or M1 (0.5 μg/mL) at 4 °C overnight. After blocking with 5% non-fat milk, 2-serial dilutions of immune sera were added and incubated at room temperature for 90 min. After washing in PBS supplemented with 0.05% Tween 20 (PBST), horseradish peroxidase (HRP)-conjugated sheep anti-mouse IgG secondary antibodies (1:2500, NA931, GE Healthcare Life Sciences) were added and incubated at room temperature for 1 h. After washing in PBST, 1-step ultra TMB substrates (34028, Thermo Scientific) were added and reactions were then stopped by addition of 1M H_2_SO_4_. Optical absorbance (OD_450nm_) was read in a microplate reader (Molecular Devices). Serum antibody titer was defined as the reciprocal dilution factor that resulted in OD_450nm_ that was ~3 times higher than the background values. For detection of subtype antibody titer, HRP-conjugated anti-mouse IgG1 (046120, Invitrogen, Thermo Fisher Scientific) and IgG2c (A90136P, Bethyl Laboratories) secondary antibodies were used.

### 2.6. Cellular Immune Response

To measure vaccine-specific CD4^+^ and CD8^+^ T cells in peripheral blood mononuclear cells (PBMCs), a small volume of blood (~50 μL) was collected into heparinized tubes followed by red blood cell (RBC) lysis. PBMCs were then stimulated with 1 μg/mL NP or M1 in the presence of 4 μg/mL anti-CD28 antibodies overnight. Next day, Brefeldin A (420601, BioLegend) was added 5 h before cell harvest. PBMCs were then stained with fluorescence-conjugated anti-CD4 (RM4–5) and anti-CD8 (53–6.7) antibodies, fixed and permeabilized, and then stained with fluorescence-conjugated anti-IFNγ (XMG1.2) and anti-IL4 antibodies (11B11). Cells were then subjected to flow cytometry analysis in BD FACSVerse.

### 2.7. Lethal Viral Challenge

Mouse-adapted influenza A/Puerto Rico/8/1934 (H1N1) viruses (NR-28652, abbreviated as PR8) were obtained from BEI Resources. LD50 of PR8 viruses was first determined [20]. In brief, groups of mice (*n* = 5) were infected with 10^0^, 10^1^, 10^2^, 10^3^, 10^4^, and 10^5^ TCID50 influenza viruses. Survival and body weight were monitored daily for 14 days. LD50 was calculated by the method of Reed and Muench [20]. For lethal viral challenge, mice were intranasally inoculated with 4 × LD50 of influenza viruses under light anesthesia. Body weight and survival were monitored daily for 14 days. Mice with body weight loss more than 20% were euthanized and regarded as dead.

### 2.8. Cytokine Levels

Serum IL-6 levels were measured by mouse IL-6 ELISA Ready-SET-Go kit (88-7064-88, Invitrogen). 

### 2.9. Statistics

Values were expressed as mean ± SEM (standard error of the mean). One-way analysis of variance (ANOVA) with Tukey’s multiple comparison test was used to compare differences for more than two groups, except otherwise specified. P-value was calculated by PRISM software (GraphPad, San Diego, CA, USA) and considered significant if it was <0.05.

## 3. Results

### 3.1. RFA Enhances NP-Induced Cellular Immune Responses

**Figure 1:**
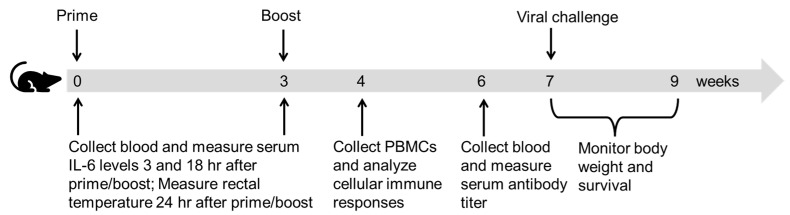
Schematic illustration of experimental design.

Mice were subjected to prime/boost immunizations of ID NP/M1 alone or in the presence of RFA, or IM NP/M1 in the presence of AddaVax, or ID PBS (Figure 1). Cellular immune responses were explored one week after boost (Figure 1). Briefly, PBMCs were isolated and stimulated with NP or M1 to evaluate percentage of IFNγ or IL4-secreting cells in CD4^+^ and CD8^+^ T cells. 

**Figure 2:**
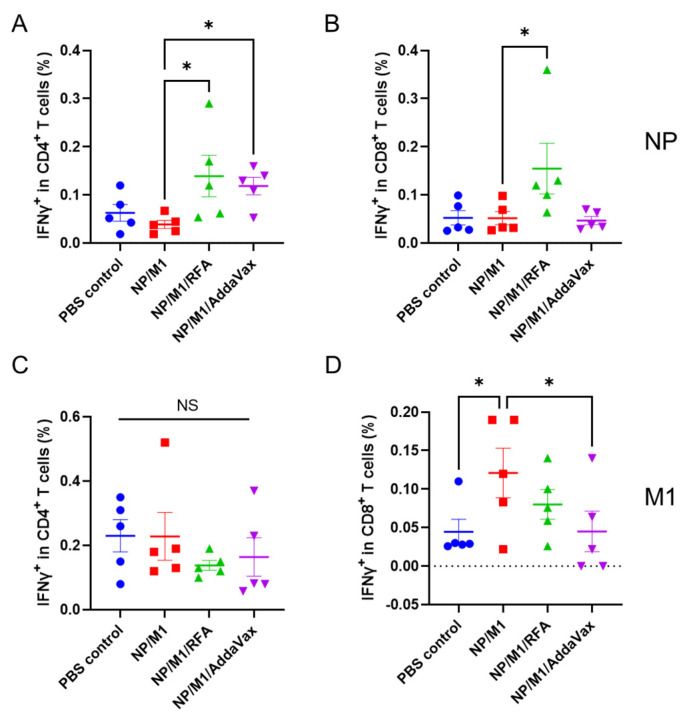
RFA enhances NP-specific cellular immune responses. C57BL/6 mice were subjected to RF or sham treatment followed by ID injection of 5 µg NP and 5 µg M1 into RF (NP/M1/RFA) or sham-treated skin (NP/M1), or IM injection of 5 µg NP and 5 µg M1 in the presence of AddaVax (NP/M1/AddaVax), or ID injection of PBS (PBS control). Immunizations were repeated 3 weeks later. PBMCs were collected one week after boost, stimulated with NP or M1 followed by intracellular cytokine staining and flow cytometry analysis. Cells were first gated based on FSC and SSC and then based on CD4 and CD8. Percentage of IFNγ-secreting cells in CD4^+^ and CD8^+^ T cells stimulated by NP are shown in (**A**,**B**) and stimulated by M1 are shown in (**C**,**D**), respectively. *n* = 5. One-way ANOVA with Fisher’s LSD test was used to compare difference between NP/M1 and other groups. *, *p* < 0.05; **, *p* < 0.01; ***, *p* < 0.001; NS, not significant.

As shown in (Figure 2A), ID NP/M1 immunization in the presence of RFA was found to significantly increase NP-specific IFNγ^+^CD4^+^ T cells as compared to ID NP/M1 immunization alone. Percentage of NP-specific IFNγ^+^CD4^+^ T cells in NP/M1/RFA group was increased by ~3.6 folds as compared to that in NP/M1 group. IM NP/M1 immunization in the presence of AddaVax also significantly increased percentage of NP-specific IFNγ^+^CD4^+^ T cells to a level similar to that in NP/M1/RFA group. We further found ID NP/M1 immunization in the presence of RFA also significantly increased NP-specific IFNγ^+^CD8^+^ T cells as compared to ID NP/M1 immunization alone (Figure 2B). Percentage of NP-specific IFNγ^+^CD8^+^ T cells in NP/M1/RFA group was increased by ~3 folds as compared to that in NP/M1 group. Interestingly, IM NP/M1 immunization in the presence of AddaVax failed to increase the percentage of NP-specific IFNγ^+^CD8^+^ T cells (Figure 2B). Interestingly, M1-specific IFNγ^+^CD4^+^ T cells showed no significant difference among groups (Figure 2C). M1-specific IFNγ^+^CD8^+^ T cells significantly increased in NP/M1 group as compared to PBS control, while significantly reduced in NP/M1/AddaVax group as compared to NP/M1 group (Figure 2D). These results indicated RFA could significantly increase NP-induced IFNγ-secreting CD4^+^ and CD8^+^ T cells. 

### 3.2. RFA Has a Minimal Effect on Humoral Immune Responses

**Figure 3:**
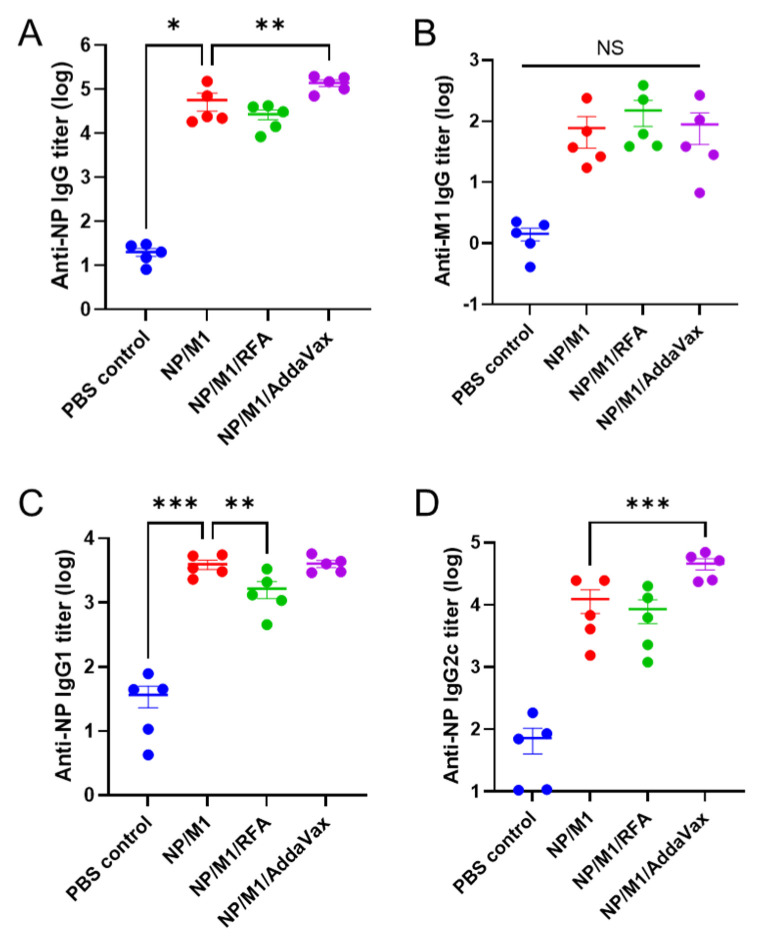
RFA had a minimal effect on NP and M1-induced antibody responses. Anti-NP and anti-M1 antibody responses were evaluated 3 weeks after boost. (**A**) Serum anti-NP IgG titer. (**B**) Serum anti-M1 IgG titer. (**C**) Serum anti-NP IgG1 titer. (**D**) Serum anti-NP IgG2c titer. *n* = 5. One-way ANOVA with Fisher’s LSD test was used to compare differences between NP/M1 and other groups. *, *p* < 0.05; **, *p* < 0.01; ***, *p* < 0.001; NS, not significant.

Although our focus is to induce internal antigen-specific CTL responses to eliminate virus-infected cells, we also compared NP and M1-specific antibody responses after boost. As shown in Figure 3A,B, NP/M1 immunization induced significant anti-NP but weak anti-M1 IgG titer. Anti-NP IgG titer was also significantly higher in NP/M1/AddaVax group than that in NP/M1 group (Figure 3A). We further evaluated anti-NP subtype IgG1 and IgG2c antibody titer. As shown in Figure 3C,D, significantly higher anti-NP IgG2c but not IgG1 antibody titer was found in NP/M1/AddaVax group than that in NP/M1 group. RFA failed to significantly increase anti-NP IgG1 or IgG2c antibody titer and rather significantly reduced anti-NP IgG1 antibody titer (Figure 3C,D). Overall, RFA showed a minimal effect on NP and M1-induced antibody responses. 

### 3.3. RFA Safely Boosts NP/M1 Immunization

**Figure 4:**
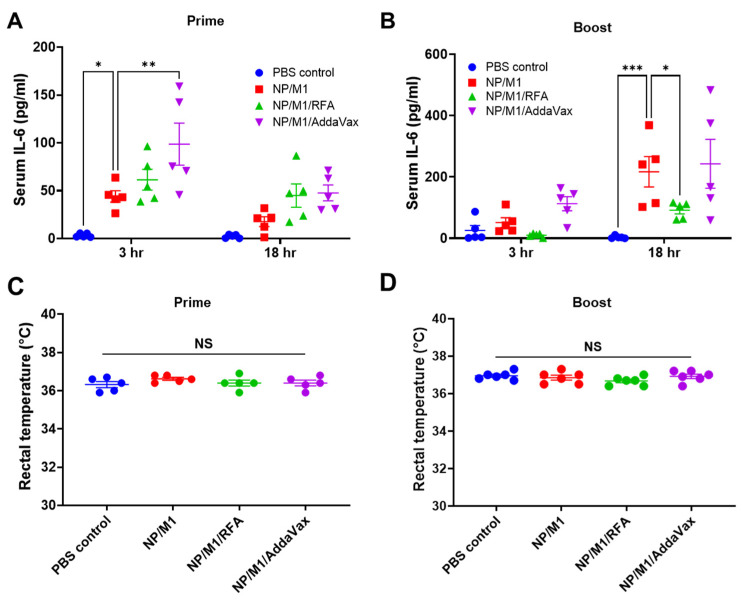
Systemic safety of the different immunizations. (**A**,**B**) Serum IL-6 levels were measured 3 and 18 h after prime and boost immunization and are shown in (**A**,**B)**, respectively. (**C**,**D**) Rectal temperature was measured 24 h after prime and boost immunization and shown in **(C**,**D**), respectively. Two-way ANOVA with Turkey’s multiple comparison test was used to compare differences between NP/M1 and other groups in (**A**,**B**). One-way ANOVA with Turkey’s multiple comparison test was used to compare differences among groups in (**C**,**D**). *n* = 5. *, *p* < 0.05; **, *p* < 0.01; ***, *p* < 0.001. NS, not significant.

Systemic safety of immunization was also explored. IL-6 and C-reactive protein (CRP) are commonly used as systemic inflammatory mediators and their serum levels are highly associated with systemic reactogenicity of vaccines in humans [21]. Due to the significant baseline serum CRP levels in mice [22], we selectively measured serum IL-6 levels 3 and 18 h after immunization. As shown in Figure 4A, prime NP/M1 immunization significantly increased serum IL-6 levels at 3 h and incorporation of RFA failed to significantly increase serum IL-6 levels. Interestingly, prime NP/M1 immunization in the presence of AddaVax induced significantly higher serum IL-6 levels at 3 h as compared to NP/M1 immunization alone. Serum IL-6 levels reduced at 18 h and showed no significant difference between NP/M1 and other groups (Figure 4A). In boost immunization, serum IL-6 levels showed no significant difference between NP/M1 and other groups at 3 h (Figure 4B). ID NP/M1 immunization significantly increased serum IL-6 levels at 18 h (Figure 4B). Interestingly, incorporation of RFA significantly reduced serum IL-6 levels (Figure 4B). Serum IL-6 levels were similar between NP/M1 and NP/M1/AddaVax groups (Figure 4B). Besides systemic IL-6 levels, we also measured rectal temperature 24 h after prime and boost immunization. Rectal temperature was measured with a mouse rectal temperature probe connected to PhysioSuite (Kent Scientific) as in our previous report [23]. We found there was no significant difference of rectal temperature among groups (Figure 4C,D). Besides systemic safety, ID NP/M1 immunization in the presence of RFA induced minimal local reactions, as observed in our previous studies [18]. Our data support the safety of RFA to boost ID NP/M1 immunization. 

### 3.4. RFA Increases NP/M1-Induced Protection against Body Weight Loss

**Figure 5:**
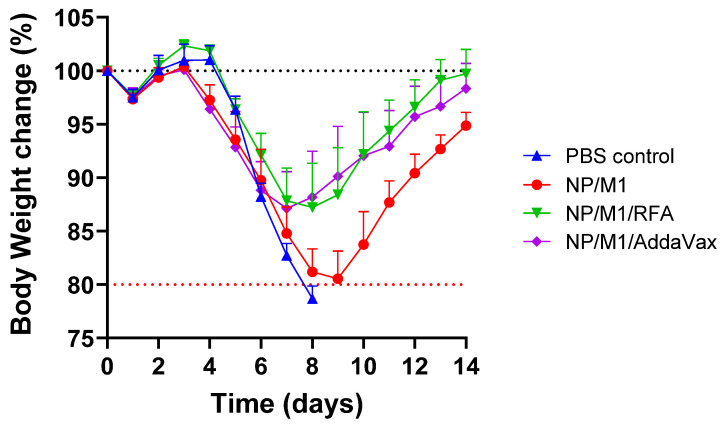
Protection against body weight loss after lethal viral challenges. Mice were intranasally challenged with 4 × LD50 of mouse-adapted PR8 viruses 28 days after boost. Body weight loss was monitored daily for 14 days. *n* = 5.

Mice were then challenged with 4 × LD50 of PR8 viruses and body weight was monitored daily for 14 days. As shown in Figure 5, mice in all groups had a similar rate of body weight loss in the first 6 days due to the lack of neutralizing antibodies. Mice in PBS control and NP/M1 groups continued to lose weight after day 6 (Figure 5). Mice in PBS control group lost more than 20% body weight on day 9 (humane endpoint). The majority of mice in NP/M1 group lost a maximal of 20% body weight on day 9 and recovered to 95% of their original body weight on day 14. In contrast, mice in NP/M1/RFA and NP/M1/AddaVax groups lost a maximal 13% body weight one week after challenge and recovered to 100% and 98% of their original body weight on day 14, respectively. As compared to PBS control, ID NP/M1 immunization in the presence of RFA and IM NP/M1 immunization in the presence of AddaVax but not ID NP/M1 immunization alone significantly reduced body weight loss on day 8 (Table 1). As compared to ID NP/M1 immunization alone, ID NP/M1 immunization in the presence of RFA and IM NP/M1 immunization in the presence of AddaVax significantly reduced body weight loss on day 9 and 10 (Table 1). 

### 3.5. RFA Increased NP/M1-Induced Protection against Lethality

**Figure 6:**
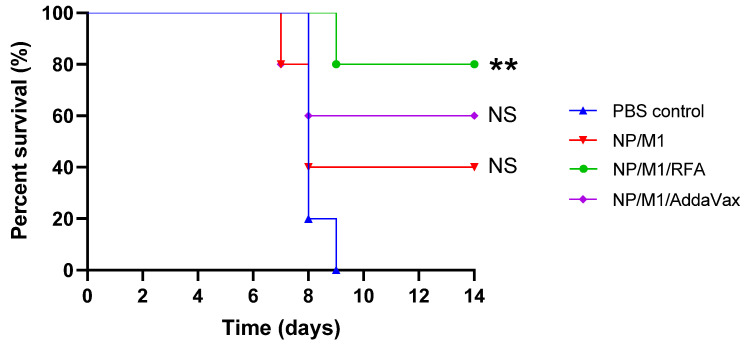
Protection against lethality after lethal viral challenges. Survival of virus-challenged mice was monitored for 14 days. Mice were regarded as dead if their body weight loss was more than 20%. *n* = 5. Log-rank (Mantel-Cox) test was used to compare differences of survival between PBS control and other groups. **, *p* < 0.01. NS: not significant.

Survival of mice after viral challenges was also explored. All mice in PBS control group died or reached humane endpoint of euthanasia within 9 days. Two out of 5 mice in NP/M1 group and 3 out of 5 mice in NP/M1/AddaVax group survived the lethal viral challenge, while 4 out of 5 mice in NP/M1/RFA group survived the challenge (Figure 6). ID NP/M1 immunization alone or IM NP/M1 immunization in the presence of AddaVax failed to significantly increase the survival of mice, while ID NP/M1 immunization in the presence of RFA significantly increased the survival of mice (Figure 6). 

## 4. Discussion

This study indicated RFA was effective to boost recombinant NP/M1 vaccination. RFA was found to significantly increase NP-specific CTL responses and NP/M1-induced protection against homologous viral challenges. External proteins are mainly presented on MHC class II molecules and elicit humoral immune responses. The induction of significant CTL responses against recombinant NP hinted RFA enabled cross-presentation of external antigens. In our previous report, we found RFA also enabled cross-presentation of OVA and rHA to induce potent CTL responses [18]. Interestingly, RFA failed to induce significant M1-specific CTL responses in this study. The underlying reason remained unknown but may reflect the uniqueness of M1 as compared to the other three antigens (OVA, rHA, and NP). For example, M1 failed to elicit potent antibody responses, while the other antigens could elicit potent antibody responses. M1 alone elicited potent CTL responses, while the other antigens alone failed to elicit potent CTL responses. In our study, we found RFA also significantly enhanced NP-specific IFNγ^+^CD4^+^ T cells. The potential role of NP-specific IFNγ^+^CD4^+^ T cells in induction of potent IFNγ^+^CD8^+^ T cells and overall protection remains to be explored. 

Our study compared relative immunogenicity and protective efficacy of ID NP/M1 immunization in the presence of RFA to IM NP/M1 immunization in the presence of AddaVax. Due to the high risk of AddaVax adjuvant to induce significant local reactions following ID delivery [24], IM route was used for delivery of NP/M1 in the presence of AddaVax adjuvant in our study. We found NP/M1 immunization in the presence of RFA significantly increased NP-specific CTL responses, while NP/M1 immunization in the presence of AddaVax significantly increased NP-specific antibody responses. Interestingly, NP/M1 immunization in the presence of AddaVax also induced significant protection against body weight loss, similar to that induced by NP/M1 immunization in the presence of RFA. The significant protection observed in NP/M1/AddaVax group was likely to be mediated by anti-NP antibody responses. In support, non-neutralizing anti-NP antibodies have been found to also confer protection against viral challenges [25]. Interestingly, NP/M1 immunization in the presence of RFA significantly increased survival of mice as compared to PBS control, while NP/M1 immunization in the presence of AddaVax failed to do so. We only challenged homologous PR8 virus where the recombinant NP and M1 were originated from. However, due to the high homology of NP and M1 sequences among influenza A viruses, we believe NP/M1 immunization in the presence of RFA may confer similar protections against other influenza A viruses, which will be explored in the near future. The relative contribution of anti-M1 immune responses to overall protection in NP/M1/RFA group will be also characterized to support the dual antigen approach. Furthermore, the duration of NP-specific IFNγ^+^CD8^+^ T cells will be also assessed to explore the ability of NP/M1 immunization in the presence of RFA to elicit durable CTL responses and long-term protection.

RFA was safe to boost vaccination. Serum IL-6 levels were used in our study as a systemic inflammatory mediator due to its close association with systemic adverse reactions of vaccines [21]. RFA failed to significantly increase NP/M1-induced systemic IL-6 release in prime immunization and rather reduced NP/M1-induced systemic IL-6 release in boost immunization. The reason that RFA reduced NP/M1-induced systemic IL-6 release remained to be explored but may reflect the uniqueness of the physical RFA to boost ID vaccination considering physical RFA briefly treats the skin without causing overt reactions. Our previous studies found RFA only induced transient low-level local inflammation, while ID injection of chemical adjuvants (Alum, MF59, MPL) induced lasting and more significant local inflammation [18]. 

Other strategies have been explored to elicit anti-NP and anti-M1 CTL responses and confer protection against influenza viral infection. These strategies include the development of DNA and viral vector vaccines and through virus-like particle (VLP) platforms [1,10,26,27,28]. As compared to these strategies, the development of adjuvants to aid recombinant NP and M1 to elicit CTL responses has the advantage that recombinant NP and M1 represents traditional vaccine type and currently we are lacking a universal VLP platform to present influenza internal antigens to elicit potent CTL responses. The physical RFA represents a promising adjuvant capable of enhancing NP-specific CTL responses. Our previous study also indicated RFA was at least comparable to CpG and AddaVax to elicit OVA and rHA-specific CTL responses [18]. Besides its high potency to induce CTL responses, RFA also has below advantages to boost vaccination. First, it does not need to modify vaccine manufacturing considering it is used to elicit tissue stress with potential release of endogenous danger signals to enhance vaccine-induced immune responses. Second, it induces minimal local and systemic adverse reactions. RFA device can also be used repeatedly for cost-effective adjuvantation. 

## 5. Conclusions

Conserved internal antigen-based universal T-cell vaccines are under development to induce cross-reactive CTL responses and confer cross-protection against influenza A viruses. Vaccine adjuvants hold a great promise to induce cross-presentation of influenza internal antigens and elicit potent CTL responses. Yet, the majority of approved adjuvants mainly enhance humoral immune responses. We took advantage of our recently developed physical RFA capable of elicitation of potent CTL responses against protein antigens to develop recombinant NP/M1-based universal T-cell vaccines. Results from the current study support the potency and safety of RFA to aid recombinant NP/M1 to induce potent NP-specific CTL responses and protection against homologous viral challenges in murine models. Our data support further development of the physical RFA and recombinant NP/M1-based universal T-cell vaccine. 

## Figures and Tables

**Table 1:** Statistical analysis of percent body weight change between groups.

	NP/M1	NP/M1/RFA	NP/M1/AddaVax	Reference Group
Day 8	NS	*p <* 0.05	*p <* 0.01	PBS control
Day 9	-	*p <* 0.05	*p <* 0.05	NP/M1
Day 10	-	*p <* 0.05	*p <* 0.05	NP/M1

(Note: Two-way ANOVA with Dunnett’s multiple comparison test was used to compare differences between groups. NS, not significant).

## Data Availability

Not applicable.
